# Microelectrode Arrays with Overlapped Diffusion Layers as Electroanalytical Detectors: Theory and Basic Applications

**DOI:** 10.3390/s131013659

**Published:** 2013-10-11

**Authors:** Peter Tomčík

**Affiliations:** Department of Chemistry & Physics, Faculty of Education, Catholic University in Ružomberok, Hrabovská cesta 1, SK-034 01, Ružomberok, Slovak Republic; E-Mail: peter.tomcik@ku.sk

**Keywords:** photolithography, interdigitated microelectrode arrays, microband electrodes, collection efficiency, redox cycling, substitutional stripping analysis, detection of catecholamines, neurotransmitters

## Abstract

This contribution contains a survey of basic literature dealing with arrays of microelectrodes with overlapping diffusion layers as prospective tools in contemporary electrochemistry. Photolithographic thin layer technology allows the fabrication of sensors of micrometric dimensions separated with a very small gap. This fact allows the diffusion layers of single microelectrodes to overlap as members of the array. Various basic types of microelectrode arrays with interacting diffusion layers are described and their analytical abilities are accented. Theoretical approaches to diffusion layer overlapping and the consequences of close constitution effects such as collection efficiency and redox cycling are discussed. Examples of basis applications in electroanalytical chemistry such as amperometric detectors in HPLC and substitutional stripping voltammetry are also given.

## 1. Introduction

In the course of research on microelectrodes, it was shown that if a characteristic dimension of a working electrode decreases to values comparable with the electrode diffusion layer thickness this offers certain advantages for electrochemical measurements [1,2], including high density steady-state diffusion flux of electroactive species towards the microelectrode surface [3,4] and low capacity of the electrical double layer [5,6]. Current also measurable in two electrode arrangements with a small amount of supporting electrolyte [7,8] and high signal to noise ratios [9,10] make microelectrodes efficient devices for applications in trace analysis [11,12] and microanalysis [13,14]. Current responses, however, are relatively low due to the small microelectrode area [15,16] causing problems with measurement of the microelectrode response [17].

One way to overcome this main disadvantage of microelectrodes while retaining their advantageous properties is coupling them in arrays. Microelectrode systems with single microelectrodes placed at a very small distance from each other allowing for the overlap of their diffusion layers are the subject of intensive research. Close geometrical constitution is a starting point for some effects bringing significant benefits in the electroanalysis of electroactive and electroinactive species. These effects can be achieved not only by a polarization of each electrode in the array with the same potential, but also by a polarization of individually addressable microelectrodes with various potentials performed by a multipotentiostat.

Electroactive species generated on one microelectrode (the so called generator segment of the array) may diffuse across the small gap towards the second microelectrode (the collector segment of the array) where they can be electrochemically detected. Like in the case of the rotating ring disc electrode, a collection efficiency parameter can be defined. Substantially higher values can be achieved for microelectrode arrays with suitable geometry due to non-convective mass transport to the microelectrode array surface. If the original analyte is formed on the collector, it diffuses back to generator due to its concentration deficit and may oxidize/reduce again, causing a redox cycling effect that enhances current without capacity noise, thus increasing the sensitivity and selectivity of the electrochemical analysis.

## 2. Theory of Mass Transport at Microelectrode Arrays

Theoretical [18] studies are focused on chronoamperometry [19] and linear sweep voltammetry [20] at microelectrode arrays consisting microcylindrical and microband electrodes [21,22]. For the diffusion layer thickness δ of a microband electrode [23] the following equation can be derived:

(1) $$\delta =\frac{\text{zFADc}}{\text{I}}$$

where z—number of electrons taking place in electrode reaction; F—Faraday constant; A—microelectrode area; D—diffusion coefficient; c—concentration of electroactive species in bulk phase of analyzed solution; I—diffusion current. Due to small characteristic dimensions of the microelectrode, however, a spherical (nonlinear diffusion) should be taken into account, therefore the diffusion current can be expressed as:

(2) $$\text{I}=\frac{{\text{zFAcD}}^{1/2}}{\sqrt{\pi \text{t}}}+\frac{\text{zFcDP}}{2}={\text{I}}_{\text{Cott}}+\frac{\text{zFcDP}}{2}$$

where t is duration of experiment, I_Cott_ is current calculated from Cottrell's equation and P is a parameter dependent on the microelectrode geometry. Very important characteristic parameters for a microband electrode are its width W_e_ and length L. Considering those facts, Aoki [23] has derived an equation for the diffusion layer thickness of a microband electrode in a dimensionless time domain:

(3) $$\Theta =\frac{\text{Dt}}{{\text{W}}_{\text{e}}^{2}}<10^{8}$$

where W_e_ is microelectrode width. In this time domain diffusion layer width is comparable with the electrode characteristic dimensions [24].

(4) $$\delta =\frac{{\text{W}}_{\text{e}}}{\frac{1}{\sqrt{\pi \Theta }}+0.97-1.10\exp\;\left[\frac{-9.90}{\left|\ln(12.37\Theta )\right|}\right]}$$

From this equation it is apparent that diffusion layer width is growing with time and is lower than that calculated from Cottrell equation due to the edge effect [24]. For very short times the diffusion layer thickness becomes equal to that calculated from the Cottrell equation. When the time is sufficiently long, a steady-state current is reached and a voltammogram recorded at a low scan rate will have a sigmoidal shape [25]. Diffusion layer thickness grows with microelectrode width W_e_ [26]. When the microelectrode is thinner (low W_e_), the time change of δ is higher and shorter times are needed to obtain steady-state currents [27].

### 2.1. Diffusion Layer Overlapping at Microband Arrays of Electrodes

**Figure 1.**
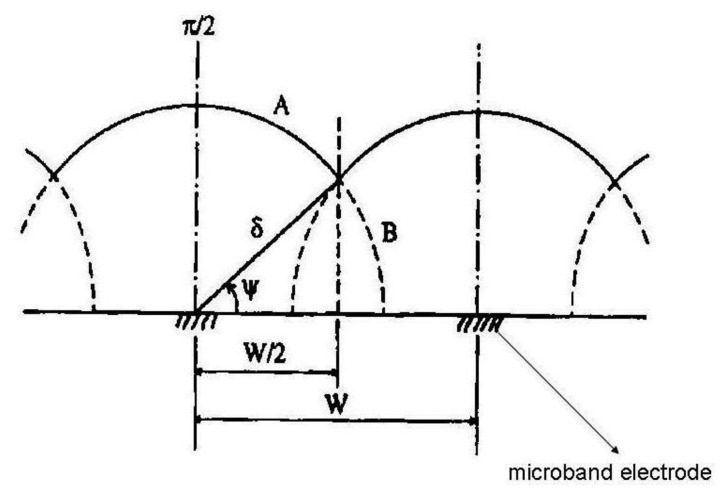
Cross-section model of diffusion layer overlap for a microband electrode [28].

For the pictorial description of the overlapping diffusion layers of an array containing microband electrodes, three cases should be distinguished:

1. When δ < W/2 where W = W_e_ + W_g_ and W_g_ is the gap width between two adjacent microelectrodes, there is no diffusion layer overlap and the total current of the microelectrode array is equal to the sum of the currents of all microelectrodes coupled in the array:
  (5) $${\text{I}}_{\text{total}}=\text{mI}$$
  where m is number of single electrodes in the array and current I can be calculated from Equation (2).
2. When δ > W/2 diffusion layers of two adjacent microelectrodes are partially overlapped. Ju [28] suggested for quantitative consideration of microband electrodes overlapping angle Ψ (Figure 1):
  (6) $$\Psi =\tan^{-1}\left(\frac{{\left[\delta^{2}-{\left(\text{W}/2\right)}^{2}\right]}^{1/2}}{\text{W}/2}\right)$$
  For long electrolysis times the angle Ψ and diffusion layer thickness are growing, however the microelectrode area is lower, therefore the magnitude of the diffusion current is lower in comparison with case 1. Equation (7) offers good results in a time domain Θ < 10^8^ in which time dependent two dimensional diffusion is taken into account and additionally the microelectrode has a sufficient length, so as to not consider the edge effect on both its ends:
  (7) $${\text{I}}_{\text{total}}=\text{mzFDcL}\left[\frac{1}{\pi \Theta }+0.97-1.10\exp\left(-\frac{9.90}{\ln(12.37)}\right)\right]\text{K}$$
  where:
  (8) $$\text{K}=1-\frac{2}{\pi }\tan^{-1}\sqrt{\frac{4{\text{W}}_{\text{e}}^{2}}{{\left[\frac{1}{\sqrt{\pi \Theta }}+0.97-1.10\exp\left(-\frac{9.90}{\left|\ln(12.37)\right|}\right)\right]}^{2}\text{W}-\frac{{\text{W}}^{3}}{4}}}$$
3. If the angle Ψ reaches a limiting value Ψ = δ/2, diffusion layers are totally overlapped. For total current an equation has been derived:
  (9) $${\text{I}}_{\text{total}}=\frac{\text{mzFcD}({\text{W}}_{\text{e}}+{\text{W}}_{\text{g}})\text{L}}{\sqrt{\pi \text{Dt}}}$$
  where L is the length of one microelectrode in the array.

The quantification of diffusion layers overlap could also be described by an overlapping factor:

(10) $$\text{S}=1-\frac{{\text{I}}_{\text{total}}}{\overset{\text{m}}{\underset{\text{j}=1}{\text{∑}}}{\text{I}}_{\text{j}}}$$

where I_j_ is the current of the electrode j when others are disconnected from the multipotentiostat. The overlap factor usually represents a ratio of the diffusion layer area of an overlapped electrode to the whole area of diffusion layers of other individually addressable microelectrodes in the array. It depends on time (experiment duration) as well as on the geometrical arrangement of microelectrodes in the array. Interdigitated microelectrode arrays (IDA) have more advantageous geometry in comparison to microband electrodes [29–31]. Chronoamperograms of various types of IDA have been measured using ferrocene in acetonitrile solution as test system.

### 2.2. Chronoamperometric and Voltammetric Studies on Interdigitated Microelectrode Arrays

Generator current in dual (bipotentiostatic) mode reaches steady state in 100 ms, the time needed for species to diffuse through the gap to create a concentration gradient. Shorter times for reaching a steady state current of 10 ms can be obtained in the case where IDA microelectrodes with sub-micrometric gaps are used [32], which is important in the analysis of compounds with short lifetimes. A second advantage of IDA microelectrodes in dual mode is that chronoamperograms measured on the collector do not contain any capacity current, which represents a noise. The equation of the chronoamperometric curve was derived by Aoki [33,34] and Coen [35] based on these prerequisites:

1. Number of metal microbands of IDA array is sufficiently high to not count the edge effects;
2. The width of each metal microband W_e_ is the same;
3. The metal layer length L is many times greater than W_e;_
4. The transport of electroactive species towards the IDA microelectrode surface ocurrs by diffusion only;
5. Diffusion coefficients are equal for both redox forms of analyzed electrochemical system.

The current is similar as in the case of microband electrode function of dimensionless Aoki's time Θ:

(11) $$\frac{\text{I}}{\text{LzFDc}}=\frac{5.553}{\ln(4\Theta )}-\frac{6.791}{\ln{\left(4\Theta \right)}^{2}}$$

**Figure 2.**
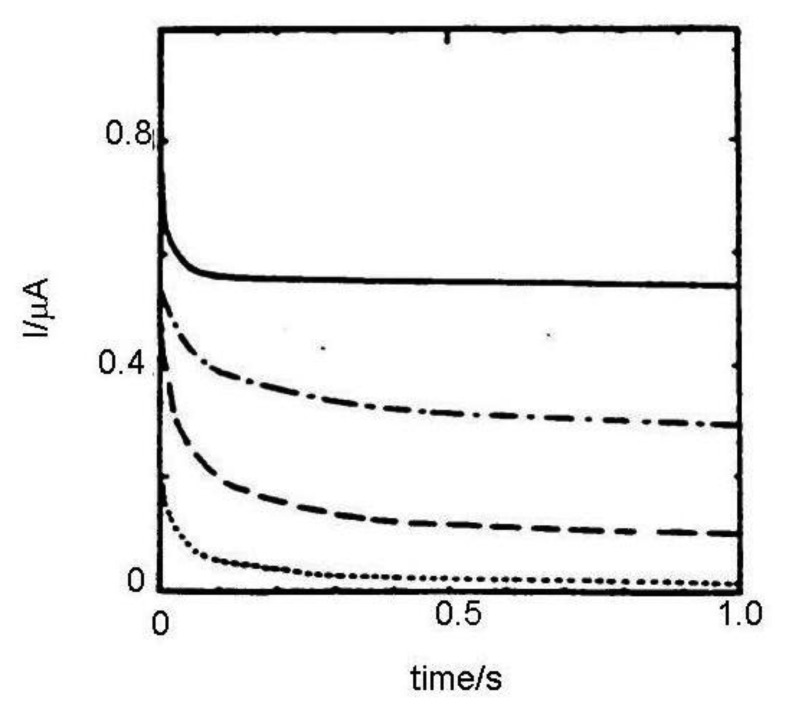
Chronoamperograms of 1 mmol·dm^−3^ ferrocene solution in acetonitrile with ammonium tetrafluoroborate on IDA array containing 50 pair of microbands with a gap of 2 μm and metal layer width of 3 μm. [36]. All results were normalized by dividing the number of microbands, (—) generator current in dual mode, (— —) generator current in single mode, (^—.—.—.—^} calculated from Aoki–Coen equation, (……) calculated from Cottrell equation.

In Reference [36] results calculated from Equation (11) and Cottrell equation were compared with chronoamperograms measured in dual and single (monopotentiostatic) mode on IDA electrodes with a microband width of 3 μm and gap of 2 μm (Figure 2). Currents were divided by the number of metal microbands and normalized by the current of single microbands. It was shown that current in single mode is higher than the current calculated from the Cottrell equation, however it is lower than the current calculated from Equation (11) due to the shielding effect [34]. Current in dual mode is higher than that calculated from the Aoki-Coen equation because of redox cycling. When the gap between two adjacent metal microbands in IDA microelectrode is increased, the current in single mode will be higher because the diffusion layer overlap is decreased, but in dual mode current it is decreased due to lower redox cycling.

**Figure 3.**
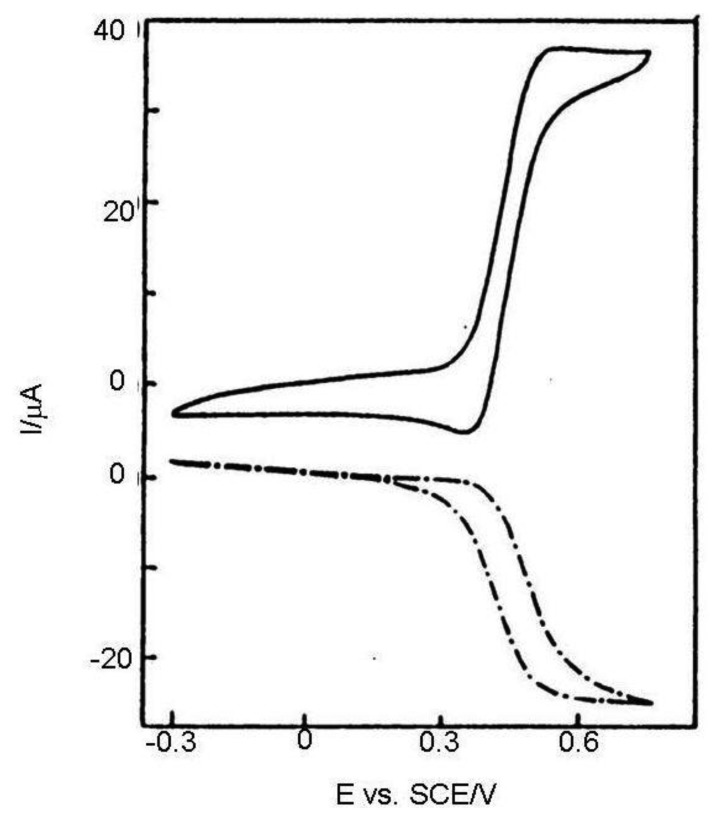
Generator and collector cyclic voltammogram at the same IDA and solution as on Figure 2 at 10,000 V/s [36].

In a classic voltammetric experiment capacity current grows with a scan rate and is faradayic with its square root. At high scan rates the voltammetric signal becomes unclear due to the high charge current (Figure 3). It can be subtracted, but the signal is deformed by the ohmic drop. This problem may be solved by an IDA microelectrode with a short time response allowing extremely short electrolysis during cyclic voltammogram recording and investigation of fast kinetics processes.

### 2.3. Collection Efficiency and Redox Cycling as Consequences of Microelectrode Array Diffusion Layer Overlap

Collection efficiency is usually calculated as a collector to generator current ratio. Bard *et al.* [36] showed that the collection efficiency depends above all on the gap between collector and generator segment of each microelectrode array and can be expressed by the equation:

(12) $$\Phi =0.095+0.33\log{\text{j}}_{\text{gap}}-0.035{\left(\log{\text{j}}_{\text{gap}}\right)}^{2}$$

where j_gap_ = 4Dt/(W_g_)^2^.

Collection efficiencies of microelectrode arrays with various geometrical arrangements were compared. On the basis of this comparison following conclusions could be summarized:

1. Collection efficiency depends not only on the gap between two individually addressable segments of microelectrode array, but also on the microelectrode width.
2. For various electrochemically reversible species with various diffusion coefficients very small changes in collection efficiency were observed, therefore collection efficiency is crucially influenced by geometrical factors.

Linear dependence of the parameter W_e_/4 + W_g_ on the square root of time (chronoamperogram duration) was observed for the case when the current reaches 50% of the limiting diffusion current. The above mentioned parameter represents an average diffusion length between generator and collector and is very suitable for evaluation of the dependence of collection efficiency on geometrical factors, especially for IDA microelectrode arrays [37].

These facts are analogous to the rotating ring-disc electrode (RRDE) used in electroanalytical chemistry for detection of electroinactive species by diffusion layer titration. The analog to an inverse value of W_g_ is the angle rate ω. Current response of RRDE is enhanced by an analyte convection during hydrodynamically stable conditions. Collection efficiency on the ring is usually less than 50% and is substantially lower than for vertically separated IDA microelectrode arrays reaching at least 95% or higher. An additional disadvantage of RRDE *vs.* IDA is the high noise related to brush contacts. Another substantial difference between RRDE and IDA is the interrelationship of diffusion layers. In the case of RRDE only the diffusion layer of the ring is influenced by the disc electrode reaction due to the centrifugal movement of analyte in close vicinity to the rotating body of the electrode, therefore the ring will never serve as a generator [38–40].

The second interesting feature of microelectrodes with interacting diffusion layers is redox cycling [41]. This phenomenon occurs when one microelectrode (the generator) is electroactive and an electrochemically reversible redox species is generated. After diffusion across the gap it is amperometrically detected on the second microelectrode with a potential corresponding to the opposite electrode reaction to the generator one (dual mode). Generator and collector currents are both in steady state and the generator current is several times higher as in the case when the collector microelectrode is disconnected from the potentiostat (single mode). This is also called forced redox cycling. An unpotentiostated microelectrode in close vicinity to the generator does not influence its response and behaves as an insulator. When a linear sweep voltammogram is recorded at very slow scan rates (less than 5 mV·s^−1^) a quasi-steady state wave is obtained, however its height is several times lower in comparison to when a suitably polarized collector microelectrode is used.

**Figure 4.**
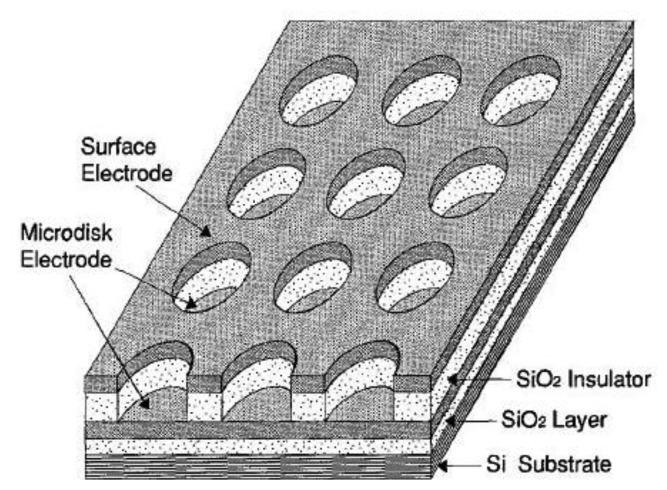
Microdisc array with vertically separated macroelectrode [42].

If a macroelectrode with significantly greater area is placed into the diffusion layer of a microelectrode which generates an electrochemically reversible species a self-induced redox cycling occurs. The macroelectrode current is high, however the diffusion layer of the microelectrode reaches only a small portion of the macroelectrode, therefore its current change is small and problematically measurable. Microelectrode current is substantially enhanced due to redox cycling between a polarized microelectrode and a polarized macroelectrode. Surprising results were obtained on the microelectrode while the macroelectrode was disconnected from the bipotentiostat. Current on the microelectrode is practically the same as measured with a polarized macroelectrode. The mechanism of self-induced redox cycling was explained by Horiuchi using a combined twin chip with microdisc array electrode (Figure 4) and a macroelectrode with the same area [42].

**Figure 5.**
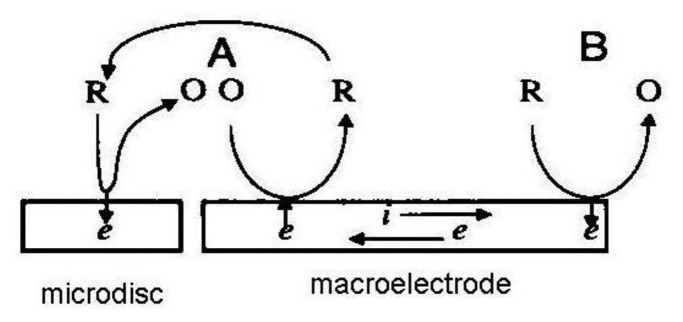
Mechanism of self induced redox cycling [42].

The current on the microdisc array electrode is higher than on the same microelectrode placed on a chip without macroelectrode. The oxidized form produced on the microdisc electrode diffuses to the vicinity of the macroelectrode and creates a local concentration gradient (point A in Figure 5). When equilibrium is established on the macroelectrode surface the opposite reaction is induced and at point B the same electrode reaction ocurrs as on the microdisc electrode. The reduced species generated on the macroelectrode at point A diffuses back to the microdisc electrode due to its concentration deficit at this location and oxidizes again, thus closing the redox cycle. Current enhancement grows with the area of the macroelectrode until it is hundred times greater than the area of the microelectrode.

A parameter for quantitative consideration of redox cycling is the number of redox cycles. It is calculated from the collection efficiencies. If collection efficiency in the direction from generator to the collector is Φ_1_ and in the opposite direction from collector to the generator it is equal to Φ_2_, the number of molecules of a species obeying one electron electrode reaction diffusion on the collector is equal to NΦ_1_ and number of molecules diffusing back to the generator is therefore NΦ_1_Φ_2_ and the number of molecules diffusing to the bulk phase of the solution is then equal to N − NΦ_1_Φ_2_ where N is number of molecules oxidized per time unit on the generator. Based on these ideas the number of redox cycles can be expressed as:

(13) $${\text{R}}_{\text{C}}=\frac{\text{N}}{\text{N}(1-\Phi_{1}\Phi_{2})}=\frac{1}{1-\Phi_{1}\Phi_{2}}$$

Accuracy of parameter the R_C_ depends on the accuracies of determination of the collection efficiencies in both directions, however in the most of studies the generator and collector were the same. The average number of redox cycles can also be calculated as the current ratio of a generator in dual mode I_g_ and in single mode I_0_. The parameter I_g_/I_0_ is highly reproducible and may substitute for R_C_ values calculated from Equation (13) in the collection efficiencies range of 10%−99%.

## 3. Fabrication of Microelectrode Arrays with Interacting Diffusion Layers

**Figure 6.**
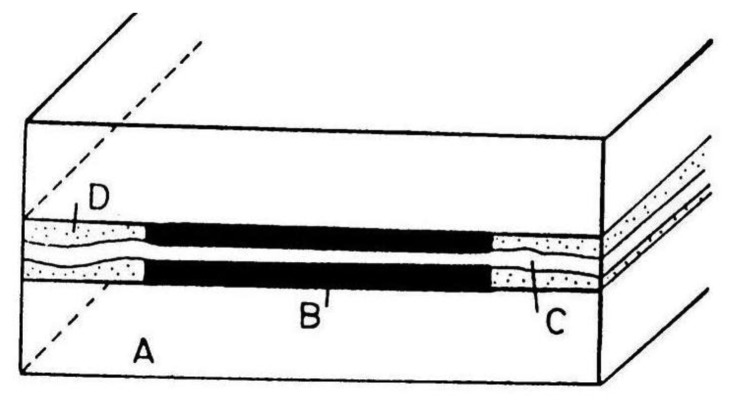
Double microband electrode: A—Glass, B—metal foil, C—spacer, D—epoxy [43].

Microband electrodes (Figure 6) are the simplest type of microelectrode arrays with interacting diffusion layers. They are fabricated by placing insulating foil of width 2, 4 or 6 μm between Pt foil with width of 4 μm on a glass wafer [43]. The construction was improved by adding a small amount of epoxide on the glass wafer and between all foils. The electrode surface was cleaned by sonication in a mixture of methanol and water. The electrode length was determined optically.

Ferocene in acetonitrile solution served as a testing redox system. Collection efficiency calculated as collector *vs.* generator current ratio was in the range of 10% to 50% and its value does not depend on ionic strength of the solution [44–46].

A very efficient fabrication technique for microelectrode arrays is photolithography, widely used in book printing, gravure printing, offset techniques and microelectronics. The most important field of photolithography is planar technology for the fabrication of planar microelectronic elements like integrated circuits, *etc.* In this technique a silicon wafer covered with a thin layer of silicon dioxide is used. A continuous metal layer is placed on this wafer and final shaping is performed by photolithographic etching. By using photoresists a horizontal relief is formed in dielectric and metal layers on the surface of silicon semiconductor. Electron resists are mostly used for microelectrode array fabrication because relief etching is performed by a flux of electrons, X–rays and reactive ions. Photoresist exposition is a diffusion process based on insolubility of cross-linked (illuminated) polymers. During photoresist exposition the dark part of the photoresist is dissolved in hot solution of the developer, leaving uncovered parts of SiO_2_ on the wafer surface and the illuminated (cross-linked) part of the photoresist will remain. Uncovered parts are etched by using a photomask for obtaining the desired geometrical shape. Finally, a metal layer is incorporated into the etched places [47–49]. For microelectrode array fabrication process chemically cross-linked photoresists are successfully used [50–53]. Their work is based on diffusion of acid protons catalyzing cross-linking process into the resist. After exposition protons are reduced to H_2_ and lose catalytic ability [54].

**Figure 7.**
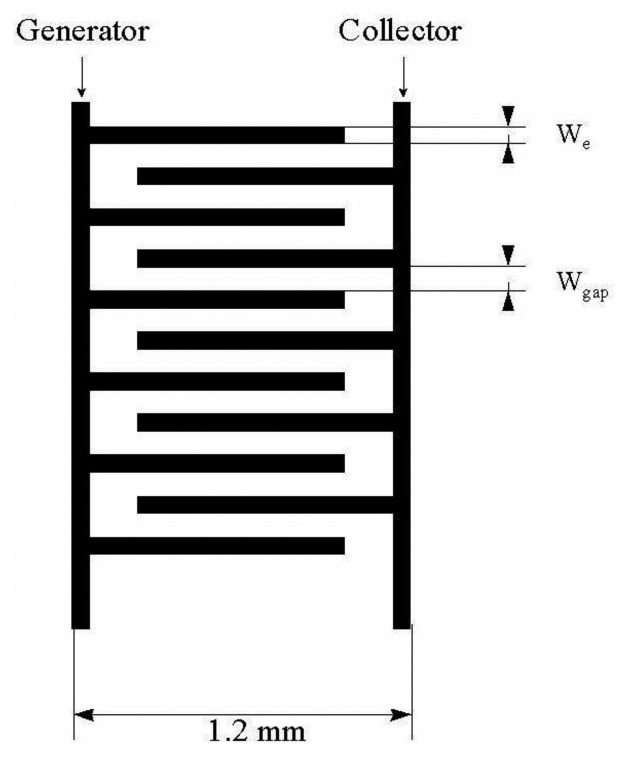
Horizontally separated IDA array [55].

**Figure 8.**
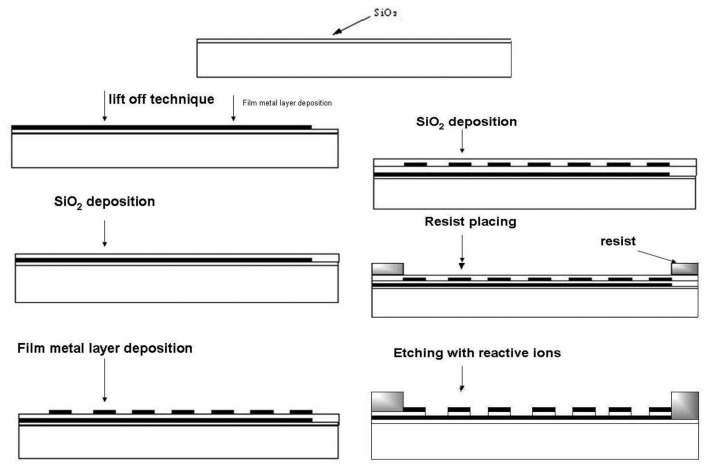
Scheme of vertically separated IDA array photolitographic fabrication.

Horizontally (Figure 7) and vertically (Figure 8) separated IDA microelectrodes were fabricated photolitographically. On a thermally oxidized silicon wafer a layer of photoresist was placed and exposed by UV rays through the photomask, then rinsed with deionized water, followed by Pt film placement with a width of 100 nm. Silicon dioxide film was etched with reactive ions in tetrafluoromethane [32,41]. During characterization a five times greater signal on a vertically separated IDA array with a submicrometrical gap of 0.5 μm (R_C_ = 35) was obtained in comparison with a planar IDA microelectrode (R_C_ = 7) with a gap of 2.5 μm corresponding to a high level of redox cycling [41].

It was also shown that carbon [56–58] may serve as an electrode material for IDA microelectrode fabrication. In [58] vertically separated IDA microelectrodes with a double layer of carbon and platinum are described. Fabrication of carbon film is based on pyrolysis of 3,4,9,10 perylene-tetracarboxylic acid dianhydride (PTCDA). For obtaining carbon films with large area and smooth surface slow evaporation of PTCDA is needed. The temperature of pyrolysis is about 1,000 °C to ensure good carbon film conductivity. The structure of the carbon film was checked by X-ray diffraction and Raman spectrometry. Cyclic voltammogram shows a relatively high potential shift corresponding to a high IR drop. It may be decreased by using IDA microelectrodes with combined microelectrode segments (one carbon and a second of platinum). High background current is caused by a high roughness of carbon film surface (average thickness of 5 nm).

## 4. Basic Electroanalytical Applications of Microelectrode Arrays with Interacting Diffusion Layers

Microelectrode arrays with interacting diffusion layers are very useful potential tools in contemporary electroanalytical chemistry. They can be used as sensors for fast, sensitive and highly selective determination of species which are important in biology, food, medicine and the environment. Electroanalytical voltammetric techniques based on using microelectrode arrays with interacting diffusion layers utilize effects caused by a close geometrical arrangement of individually addressable segments in the array. Redox cycling on the adjacent segments is able to enhance the current response, which may increase the sensitivity. The selectivity is enhanced by the fact that electrochemically irreversible redox pairs are not transferred on the collector [59]. Certain limitations concern usability only in the case of reversible or quasireversible charge transfer of the target species as well as the fact that a chip with a microelectrode array must be placed in non-aggressive media to avoid its damage. The safe pH range is shown to be from 3 to 11 [60]. One way to overcome these limitations is surface modification of microelectrode arrays with various polymeric films [61–63].

### 4.1. Detection of Catecholamines with IDA Microelectrodes

Short time response and current without capacity noise is utilized for amperometric detection of species in a flow system [64]. Amperometric detection in liquid chromatography is very popular in the analysis of trace amounts of organic species due its high sensitivity, selectivity [65], in addition if a microelectrode is used as amperometric detector only small amounts of the supporting electrolyte are involved. In the eighties and nineties of the last century a series of individually polarizable electrodes started to be used for this type of analysis [66–68]. However, there was no diffusion layer overlapping due to the large distance between each other, therefore they were substituted by microelectrodes with close geometrical arrangement of individually addressable segments [69,70]. The simplest type of this kind of microelectrode arrays was the double rectangular electrode (DRE) [71]. DRE has the ability to enhance the selectivity of detection when on one rectangle the interferents are irreversibly oxidized and the chromatogram obtained on the second rectangle with a more negative potential as the first one does not contain peaks of irreversible interferents. This situation is also practically the same for IDA microelectrodes [72–74], however its geometry allows one to reach substantially higher values of collection efficiency as well as higher level of redox cycling, thus enhancing current response [75–79]. This methodology is applied to the selective detection of catecholamines [80–87]. Catecholamines are important biological compounds mediating a nerve perturbation in a synaptic cleft, therefore their sensitive and selective detection is needed. Biological samples contain a significant amount of interferents [88,89] such as L-ascorbic acid (AA), uric acid (UA) and catecholamine metabolites like dihydroxyphenylacetic acid (DOPAC) and homovanilic acid (HVA) so it is important to eliminate the influence of these interferents on the sensitivity and selectivity of catecholamine determination. Collection efficiencies for catecholamines are equal to 0.53–0.56 and collection efficiencies of interferents are practically zero due to their electrochemical irreversibility. Consequently, peaks of catecholamines will become dominant in a chromatogram recorded at the collector. It was observed that steady state current on IDA microelectrode in dual mode depends on the cubic root of the flow rate. This fact is caused by redox cycling [90–92]. When an IDA microelectrode with a gap around of 1 μm is used its response is not dependent on flow rate. Further improvement of the detection limit of the determination of catecholamines results from the use of carbon IDA microelectrode arrays [93–99]. Before measurement a carbon IDA microelectrode must be electrochemically pretreated to assure a reversible response to catecholamines [100–103], however capacity current is increased and the potential window will become narrower after pretreatment [104,105]. If a carbon IDA electrode with some hundreds of generator–collector pair segments is used collection efficiency for catecholamines may increase to 70%. Due to the high level of redox cycling and hence high signal to noise ratio a detection limit around 10^−11^ mol·dm^−3^ may be reached. This corresponds to 5 fg resp. 32 amol of dopamine (absolute amount) [106,107].

IDA microelectrodes avoid the disadvantages of classical electrochemical detection such as reactions of several species on the electrode [108] and high background current. Electrochemical detection is faster than fluorescence detection based on the derivatization with 1,2-diphenylenediamine (DPE) which involves long reaction times [109–111] and other operations [112] as well as chemiluminiscence detection [113–115].

**Figure 9.**
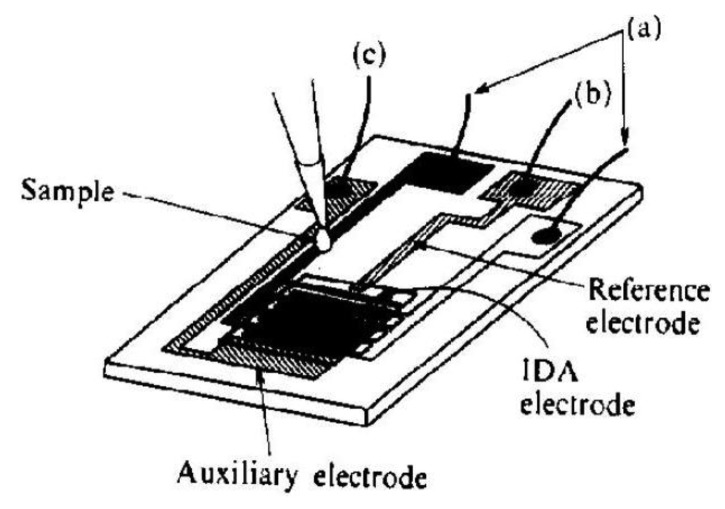
Small volume IDA electrochemical microcell [116]: (**a**) contacts for IDA microelectrodes; (**b**) contact for reference electrode; (**c**) contact for counter (auxiliary) electrode.

Another approach in the improvement of the selectivity of catecholamines determination is the use of electrochemical cells with a small volume. Microcells are constructed by lithographic placement of the sensor with 50 pairs of the segment with a width of 3 μm and 2 or 5 μm gap distance onto a silicon wafer together with reference and counter microelectrodes (Figure 9) [116]. Ascorbic acid was used as interferent. The selectivity of the sensor was tested on a 4 mL macrocell and 2 μL microcell. The model sample contained dopamine with a concentration of 1 × 10^−4^ mol·dm^−3^ and ascorbic acid at a concentration of 1 × 10^−3^ mol·dm^−3^.

**Figure 10.**
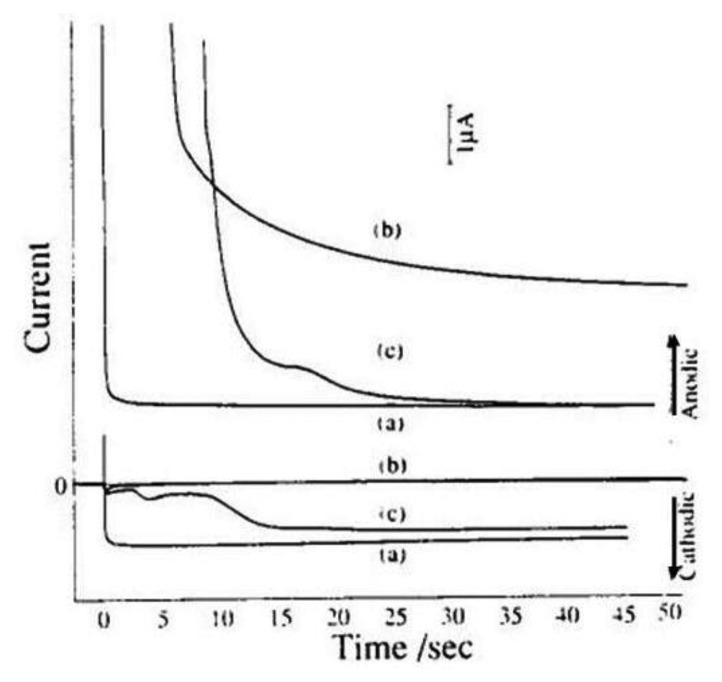
Chronoamperograms of 1 × 10^−4^ mol dm^−3^ dopamine with and without 1 × 10^−3^ mol·dm^−3^ ascorbic acid [116]. (**a**) 4 mL cell dopamine without ascorbic acid; (**b**) 4 mL cell dopamine with ascorbic acid; (**c**) 2 μL cell dopamine with ascorbic acid.

Results were compared with a dopamine signal without interferent (Figure 10). Anodic current in a macrocell with an addition of ascorbic acid is very high, corresponding its irreversible oxidation. Anodic current in a microvolume cell is also high, however in a certain time it reaches the current value of the sample without ascorbic acid due to its bulk phase (coulometric) oxidation because sensor and sample volume have comparable dimensions.

Lowering the analyzed volume increases the selectivity of electrochemical detection of catecholamines. Collection efficiency of dopamine on the concentration of ascorbic acid was investigated. It was shown in the case of macrocell decreasing collection efficiency occurred when the concentration of ascorbic acid is increased. However collection efficiency in a microvolume cell remains stable around more than 50%, even when the concentration of ascorbic acid is 30 times higher than the concentration of dopamine [116,117].

### 4.2. Electrochemical Stripping Voltammetry of Reversible Redox Species with Self Induced Redox Cycling

The lowest detection limit of all electroanalytical techniques corresponds to electrochemical stripping analysis [118–120]. It is widely used for determination of metal ions and halides in water, food, biological fluids and environmental samples [121–123]. The detection limit of electrochemical stripping analysis is usually found in order of 10^−1^–10^−11^ mol·dm^−3^ [124–126]. However this technique can only be applied to certain species such as metal ions, halides or adsorptive organic compounds by using special techniques such as chemical modification of the working electrode surface or adsorptive complex formation [127–131]. Substitutional stripping voltammetry by using an interdigitated microelectrode array as working electrode extends the usability of stripping analysis to species for which deposition onto an electrode surface is not possible due to various reasons. The common reason is the solubility of both forms of the target redox analyte.

**Figure 11.**
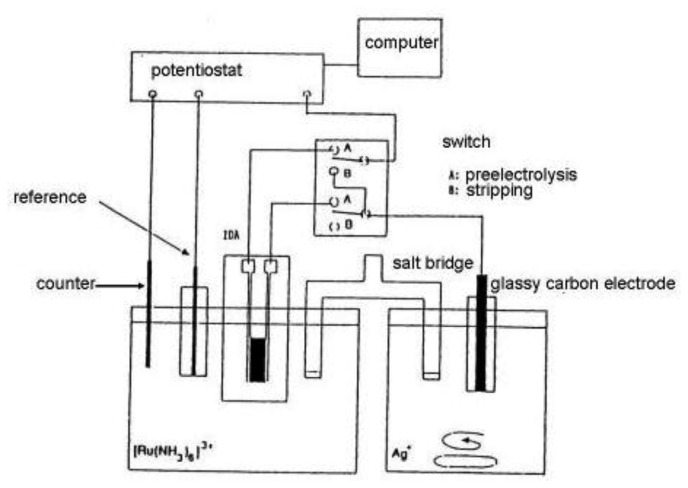
SSV measuring system scheme [132].

Substitutional stripping voltammetry (SSV) also allows detection of reversible species where both forms of the redox system are very soluble. Self-induced redox cycling as described when explaining the interaction of diffusion layers of two closely spaced electrodes may also be realized in a double compartment cell (Figure 11) [132].

In this experimental arrangement a pair of individually addressable microelectrode segments forming an IDA microelectrode is placed in the first compartment and a macroelectrode in the second one. One of pair of microelectrodes is connected with the macroelectrode. In this system a charge produced by self-induced redox cycling in the first compartment is transferred into another electrochemical reaction in the second compartment.

**Figure 12.**
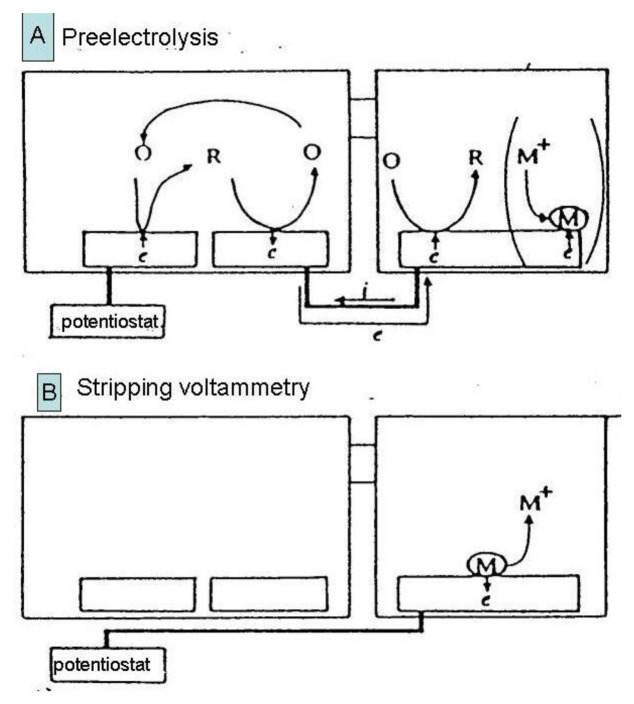
Principle of substitutional stripping analysis [132]. A—transfer of redox cycling to the right compartment during preelectrolysis step; B—electrochemical stripping of metal deposited on macroelectrode.

In the case depicted in Figure 11, an oxidized form of a reversible redox system is analyzed. Redox cycling in the first (left) compartment may induce in the second (right) compartment deposition of a substitutional compound as a solid species onto the macroelectrode surface [132,133]. This process is called preelectrolysis and its duration depends on the operator. The amount of substitutional species is determined by its stripping like in a classic voltammetric stripping analysis (Figure 12). It is proportional to the concentration of the oxidized form of the target species in the first compartment and a stripping peak is then suitable for its quantification. The term substitutional stripping voltammetry is derived from a fact that during preelectrolysis a substitutional species is deposited in the second compartment of the measuring system and stripped instead of analyzed as a reversible species with both redox forms being soluble in the solvent [133].

Substitutional stripping voltammetry has two variants. The first one is cathodic SSV in which the analyzed species is ferrocenylmethyltrimethylammonium bromide (aq–ferrocene) [133]. As a substitutional electrolyte iodide was used, together with a silver electrode in the second compartment. During preelectrolysis AgI is deposited onto the silver electrode surface offering a stripping peak at the potential of −0.2 V *vs.* SCE. Potential of the left electrode was fixed at 0.55 V *vs.* SCE corresponding to the limiting current of the oxidation to aq-ferricene. The silver electrode was polarized by a scan in the range of 0.25 to −0.4 V *vs.* SCE during the stripping step.

If the concentration of iodide is equal to 1 × 10^−6^ mol·dm^−3^ the stripping peak height is proportional to the preelectrolysis time and no stripping peak was observed in the absence aq-ferrocene. However by comparison of the charge passed during redox cycling with the stripping charge of AgI it was observed that the coulombic efficiency of charge transfer between two compartments is only 3.2%. This value is too low for assuring high sensitivity. By enhancing the iodide concentration to 1 × 10^−5^ mol·dm^−3^ practically 100% coulombic efficiency was achieved, however small stripping peaks of AgI was observed even when no aq-ferrocene is present in the first compartment because at this higher concentration of iodide deposition in the second compartment is induced by the residual current at a potential of 0.55 V *vs.* SCE in the first compartment. Approximately a 500 times greater signal is obtained compared to cyclic voltammetry.

Anodic SSV is based on the redox cycling of hexaamminruthenium(III) chloride [133]. It is the oxidized form of typically reversible redox system with a reduction potential of −0.2 V *vs.* SCE, therefore current flows in an opposite direction than in the case of aq-ferrocene. When this species is reduced on one segment of an IDA array on the second segment it is oxidized and silver ions are deposited on a glassy carbon electrode [134]. Then the silver is anodically stripped in a positive sweep of the potential.

Figure 11 depicts the experimental arrangement for anodic SSV. It happens by using a switch in a position A polarize the left segment of the IDA array working microelectrode with a constant potential of −0.4 V *vs.* SCE. At this potential reduction of [Ru(NH_3_)_6_]^3+^ to [Ru(NH_3_)_6_]^2+^ proceeds with limiting current. The right segment of the IDA array is connected with a glassy carbon electrode in the second compartment of the electrolytic cell. During preelectrolysis redox cycling of [Ru(NH_3_)_6_]^3+^ is transferred to the deposition of AgI on glassy carbon electrode in a stirred solution. After the preelectrolysis time (typically 10 min) the switch is turned to position B and the solution is stabilized during quiet conditions for 10 s. Then the potential of glassy carbon electrode is scanned from −0.4 to +0.5 V *vs.* SCE with a scan rate of 20 mV·s^−1^. The stripping peak of silver was found at +0.35 V *vs.* SCE. No peak was observed at the potential lower than −0.4 V or when no [Ru(NH_3_)_6_]^3+^ was present in a solution in the first compartment [132]. The cyclic voltammogram on the left segment of the IDA array was measured and compared with an oxidation current on the right segment of the IDA. No significant difference was observed, indicating a high collection efficiency, but oxidation current contained a substantially smaller amount of interference signals. This fundamental decrease of the background signal is the basis for preelectrolysis to assure a high signal to noise ratio achieving such high sensitivity also for the stripping peak. By comparison between cyclic voltammograms with redox cycling and silver stripping peak approximately 80 times greater signal was obtained, however this signal is 2,500 time higher than one obtained by simple cyclic voltammetry.

Otherwise, coulombic efficiency of transfer oxidation of [Ru(NH_3_)_6_]^3+^ to silver stripping was only around 43%. Detection limit was estimated to 1 × 10^−8^ mol·dm^−3^ and linear dynamic concentration range was from 1 × 10^−8^ to 1 × 10^−7^ mol·dm^−3^. Linearity was disappeared when the concentration of [Ru(NH_3_)_6_]^3+^ was increased to 1 × 10^−6^ mol·dm^−3^ [132] due to the decreasing coulombic efficiency.

As macroelectrode material for the deposition of silver ions a glassy carbon electrode was used. GC electrodee have a wide potential window and charge transfer between silver ions and its surface is reversible. Another electrodes, e.g., the hanging mercury electrode are also suitable for use in SSV experiments, however the potential window is narrower, especially in an anodic potential range thus limiting stripping potential sweeping. For assuring the sufficient deposition rate needed for charge consumption generated by redox cycling silver ions are suitable, while Hg^2+^ ions are also suitable because their potential is sufficiently higher, like the potential of [Ru(NH_3_)_6_]^3+^ oxidation. Some limitations may occur if various anions forming complexes with Hg^2+^ are present in a solution [134]. Further important parameters to be optimized are: macroelectrode area, concentration of substitutional species and scan rate during the stripping step. When the concentration of analyzed species is low, the signal to noise ratio is also low and a very small amount of Ag^+^ ions is deposited on the macroelectrode. In addition the faradayic and capacity current of various impurities remain stable therefore it is difficult to obtain a baseline on a big macroelectrode. It seems to be optimal when a glassy carbon electrode with a diameter of 1 mm is used as macroelectrode for the deposition of substitutional species. Concentration of Ag^+^ is also an important factor for decreasing the SSV detection limit. When the concentration of Ag^+^ is lower or the same as the concentration of the analyzed species in the first compartment coulombic efficiency is substantially lower than 100%, because deposition of silver ion will become the rate determining step and the whole charge produced by redox cycling during preelectrolysis cannot be transferred to the Ag^+^ deposition. Coulombic efficiency is near 100% when the Ag^+^ concentration is at least one order greater than the concentration of [Ru(NH_3_)_6_]^3+^. Similar results were obtained for cathodic SSV with iodide as substitutional species and aq-ferrocene as analyte. Scan rate during the stripping step is also an important parameter for the reliability of SSV determinations. High scan rates produce high stripping peaks with a biased baseline and it is difficult to dissolve all the silver with a linear sweep of the potential [135]. Otherwise low scan rates cause underpotential deposition of silver [136]. For both limitations a satisfactory value is 20 mV·s^−1^. Starting potential for stripping is usually set up to the potential of the generator (left) segment of the IDA array. Higher potential is also possible, however in this case current caused by switch turning from preelectrolysis mode to stripping mode may affect the stripping peak. This current does not occur if the potential of the generator segment of the IDA array and the starting potential of the stripping sweep are equal.

When SSV voltammograms of 1 × 10^−11^ mol·dm^−3^ [Ru(NH_3_)_6_]^3+^ for various preelectrolysis times were measured, stripping peaks of silver were observed at a potential of 0.3 V *vs.* SCE and they are dependent on preelectrolysis time and no peaks are obtained in the absence of [Ru(NH_3_)_6_]^3+^. This shows that electrolysis of [Ru(NH_3_)_6_]^3+^ causes the deposition of silver ions [133].

Peak height after 20 min preelectrolysis was around 0.5 nA. The theoretical value of the limiting current of the [Ru(NH_3_)_6_]^3+^ solution with a concentration of 1 × 10^−11^ mol·dm^−3^ in dual mode for the IDA microelectrode was 0.8 pA [133]. SSV signal is 630 times higher. Peak area was 1.1 nC. Charge passed during preelectrolysis was 0.96 nC, therefore coulombic efficiency is 115%. This 15% deviation may be caused by dissolved oxygen. Oxygen was removed before preelectrolysis by bubbling with pure nitrogen, however it is impossible to bubble the solution during preelectrolysis because redox cycling is very sensitive to convection. A small amount of oxygen is able to dissolve in a solution during the 20 min of preelectrolysis.

Concluding, SSV is a suitable electroanalytical technique for the detection of reversible redox species at very low concentrations. The current from the collector segment of IDA array can be integrated because it has a small background signal content. The integration may be performed by coulometer, however its resistivity may interfere with redox cycling. In SSV experiments direct connection of electrodes in various compartments minimizing internal resistivity and macroelectrode behaves as an ideal chemical coulometer.

SSV was successfully applied to the detection of dopamine. If an IDA microelectrode is used in dual mode a detection limit of 1 × 10^−8^ mol·dm^−3^ was obtained. In SSV experiments a silver macroelectrode and iodide were used for substitutional stripping. After optimization of measurement conditions a cathodic stripping peak dependent on preelectrolysis time and concentration of dopamine was observed. It was shown that it is possible to detect dopamine on a concentration level of 1 × 10^−9^ mol·dm^−3^. This detection limit is lower than on an IDA electrode in dual mode, but substantially higher than for the detection of [Ru(NH_3_)_6_]^3+^ because the detection limit of AgI stripping is about 1–2 orders of magnitude higher than anodic stripping of silver. One possibility to increase sensitivity of substitutional stripping of dopamine is by applying gold IDA microelectrodes or carbon IDA microelectrodes with quasi-reversible charge transfer of dopamine [137].

## 5. Conclusions

In this contribution microelectrode arrays with overlapped diffusion layers are described, highlighting the theory of charge transfer, discussion of close constitution effects and basic applications. These powerful electroanalytical tools can be used as very sensitive and selective detectors hyphenated with separation analytical techniques. They may recognize signals of irreversible redox systems from reversible redox species due to redox cycling in dual mode at two adjacent microelectrodes polarized with various potentials corresponding to direct and opposite electrochemical reactions taking place in electrochemical systems. Microelectrode arrays may be also used for stripping analysis of species which cannot be deposited onto an electrode surface as solid species because of their natural solubility in a solvent. Redox cycling is possible to transfer current to another compartment of an electrolytical cell inducing substitutional species deposition processes.
